# Macrophage-Associated Lipin-1 Promotes β-Oxidation in Response to Proresolving Stimuli

**DOI:** 10.4049/immunohorizons.2000047

**Published:** 2020-10-19

**Authors:** Robert M. Schilke, Cassidy M. R. Blackburn, Shashanka Rao, David M. Krzywanski, Brian N. Finck, Matthew D. Woolard

**Affiliations:** * Department of Microbiology and Immunology, Louisiana State University Health Sciences Center, Shreveport, LA 71130; † Department of Cellular Biology and Anatomy, Louisiana State University Health Sciences Center, Shreveport, LA 71130; ‡ Division of Geriatrics and Nutritional Science, Washington University School of Medicine, St. Louis, MO 63110

## Abstract

Macrophages reprogram their metabolism to promote appropriate responses. Proresolving macrophages primarily use fatty acid oxidation as an energy source. Metabolites generated during the catabolism of fatty acids aid in the resolution of inflammation and tissue repair, but the regulatory mechanisms that control lipid metabolism in macrophages are not fully elucidated. Lipin-1, a phosphatidic acid phosphatase that has transcriptional coregulator activity, regulates lipid metabolism in a variety of cells. In this current study, we show that lipin-1 is required for increased oxidative phosphorylation in IL-4 stimulated mouse (*Mus musculus*) macrophages. We also show that the transcriptional coregulatory function of lipin-1 is required for β-oxidation in response to palmitate (free fatty acid) and apoptotic cell (human) stimulation. Mouse bone marrow–derived macrophages lacking lipin-1 have a reduction in critical TCA cycle metabolites following IL-4 stimulation, suggesting a break in the TCA cycle that is supportive of lipid synthesis rather than lipid catabolism. Together, our data demonstrate that lipin-1 regulates cellular metabolism in macrophages in response to proresolving stimuli and highlights the importance of aligning macrophage metabolism with macrophage phenotype.

## INTRODUCTION

Macrophages are innate immune cells that regulate tissue homeostasis and are critical for disease resolution. Macrophages are able to polarize toward distinct phenotypes, such as proresolving macrophages that aid in wound-healing processes or proinflammatory macrophages that clear pathogens (1, 2). As macrophages polarize, they change their metabolic profile to effectively respond to stimuli (3). Proinflammatory macrophages predominately rely on glycolysis to generate ATP. Oxidative phosphorylation is reduced to generate reactive oxygen species and promote lipid synthesis (4). Proinflammatory macrophages have a break in the TCA cycle at two distinct points. The first break, a downregulation of isocitrate dehydrogenase, leads to the accumulation of citrate that is exported out of the mitochondria to be used in lipid synthetic pathways (5, 6). The second break leads to the accumulation of succinate, which stabilizes HIF1α to increase expression of proinflammatory genes. Proinflammatory macrophages also increase the pentose phosphate pathway (PPP) to generate NADPH, which is used in lipid synthesis and cellular redox potential (7). Proresolving macrophages initially increase glycolysis to quickly generate ATP but heavily rely on oxidative metabolism to produce sufficient energy to carry out their functions (7). Proresolving macrophages use β-oxidation of fatty acids to produce acetyl-CoA, which is oxidized via a series of reactions in the TCA cycle (7). The oxidation of acetyl-CoA generates reducing equivalents, such as NADH, to fuel the electron transport chain (7). β-Oxidation has been shown to promote macrophage polarization toward the proresolving phenotype and is essential to disease resolution (8, 9). Catabolism of apoptotic cell (AC)–derived lipids promotes IL-10 production and subsequent wound healing in a mouse model of myocardial infarction (8). However, how macrophages regulate lipid metabolism in response to proresolving stimuli to promote proresolving functions is not fully understood.

Lipin-1 is a phosphatidic acid phosphatase that converts phosphatidic acid into diacylglycerol to be used in lipid synthetic pathways (10). Lipin-1 also has enzymatically independent transcriptional coregulator activity in which lipin-1 binds to transcription factors, such as PPARs, to regulate their activity and subsequent gene expression (11). Lipin-1 transcriptional coregulator activity has been shown to contribute to the expression of genes that encode proteins involved in fatty acid transport and lipid catabolism in a variety of tissues and cells (12). Hepatic lipin-1 amplifies PGC-1α/PPARα regulatory pathway to stimulate β-oxidation (11). Lipin-1 not only controls fatty acid metabolism but may also regulate oxidative phosphorylation because genetic depletion of lipin-1 in skeletal muscle results in a significant decrease in oxidative phosphorylation (13). Loss of hepatic lipin-1 in mice results in an alteration in major metabolic pathways in a diurnal cycle, such as an increase in fatty acid synthesis and elevated peripheral glucose use (14). Genetic depletion of lipin-1 in *Caenorhabditis elegans* results in an increase in fatty acid synthesis genes, with a corresponding decrease in lipolytic genes (15). Individuals lacking lipin-1 have impaired β-oxidation during exercise, which can result in rhabdomyolysis (16). Collectively, these studies demonstrate the contribution of lipin-1 to lipid metabolism.

Macrophages balance their cellular metabolism to carry out their function and alterations in metabolic reprogramming prevent proper responses. Reduction in β-oxidation negatively impacts macrophage function and its consequence on tissue restoration (1, 8, 17). The two independent molecular functions of lipin-1 appear to play contrasting roles in modulating macrophage polarization. Specifically, lipin-1 enzymatic activity promotes proinflammatory macrophage responses, whereas the nuclear activity promotes proresolving properties (10, 18, 19). Lipin-1–mediated diacylglycerol production promotes the activation of the AP-1 transcription factor, which leads to the production of proinflammatory cytokines IL-6, TNF-α, and IL-12 and proinflammatory eicosanoids such as PGE_2_ (18, 19). The enzymatic activity of lipin-1 in macrophages contributes to the pathogenesis of atherosclerosis, colitis, colon cancer, LPS-induced inflammation, and alcoholic liver disease (10, 19-21). We have further demonstrated that lipin-1 can also contribute to proresolving macrophage polarization (22). Specifically, our studies suggest that lipin-1 transcriptional coregulatory activity is required for promotion of macrophage proresolving/wound-healing phenotype. Mice lacking lipin-1 from myeloid cells have accelerated atherosclerosis (C.M.R. Blackburn, R.M. Schilke, A.E. Vozenilek, B.N. Finck, M.D. Woolard, manuscript posted on bioRxiv, DOI: 10.1101/2020.06.02.130096) and a defect in wound healing, which we did not observe in mice lacking myeloid-associated lipin-1 enzymatic activity (22). However, the molecular mechanisms by which lipin-1 transcriptional coregulatory activity promotes macrophage proresolving phenotypic activity is unknown. The contribution of lipin-1 to β-oxidation in other tissues, taken together with lipin-1 involvement in polarization of macrophages toward a proresolving phenotype, led us to hypothesize that lipin-1 regulates β-oxidation in macrophages stimulated with proresolving stimuli.

We used a mouse model to genetically deplete either only the enzymatic activity of lipin-1 or completely deplete lipin-1 within myeloid-derived cells. This allows us to elucidate which activity of lipin-1 contributes to lipid metabolism within macrophages. We show that in macrophages, the transcriptional coregulatory activity of lipin-1 is required for increased oxidative metabolism in response to IL-4 and AC.

## MATERIALS AND METHODS

### Animals

All animal studies were approved by the LSU Health Sciences Center–Shreveport institutional animal care and use committee. All animals were cared for according to the National Institutes of Health guidelines for the care and use of laboratory animals.

Mice lacking lipin-1 enzymatic activity from myeloid cells (lipin-1^mEnzy^KO) were generated as previously reported (19). Briefly, mice with exons 3 and 4 of the *Lpin1* gene flanked by LoxP sites (genetic background: C57BL/6J and SV129; generously provided by B.N.F. and R. Chrast) were crossed with C57BL/6J LysM-Cre transgenic mice purchased from The Jackson Laboratory (Bar Harbor, ME). Mice fully lacking lipin-1 from myeloid cells (lipin-1^m^KO) were generated by crossing mice with exon 7 of the *Lpin1* gene flanked by LoxP sites (genetic background: C57BL/6J and SV129; generously provided by B.N.F. (23) with C57BL/6J LysM-Cre transgenic mice purchased from The Jackson Laboratory. Age-matched lipin-1 flox/flox littermate mice were used as controls.

### Generation of bone marrow–derived macrophages

Bone marrow–derived macrophages (BMDMs) were generated from lipin-1^mEnzy^KO, lipin-1^m^KO, and littermate control mice, as previously described (24). Briefly, femurs were excised under sterile conditions and flushed with D10: DMEM (Life Technologies) supplemented with 10% FBS (S11150; Atlanta Biologicals), 2 mM GlutaMAX (35050-061; Thermo Fisher Scientific), 100 U/ml penicillin-streptomycin (American Type Culture Collection), and 1 mM sodium pyruvate (HyClone). RBCs were lysed using ammonium chloride-potassium carbonate (0.15 M NH_4_Cl, 10 mM KHCO_3_,0.1 mM NA_2_EDTA, adjusted to pH 7.2 and filter sterilized in 0.22-μm filter) lysis (ACK) followed by PBS wash. Isolated cells were incubated in sterile flasks for 6 d in BMDM differentiation medium: DMEM knockout medium (11965-092; Life Technologies) supplemented with 30% L cell conditioned medium, 20% FBS (S11150; Atlanta Biologicals), 2 mM GlutaMAX (35050-061; Thermo Fisher Scientific), 100 U/ml penicillin-streptomycin (American Type Culture Collection), and 1 mM sodium pyruvate (HyClone) at 37°C and 5% CO_2_. Once cells were 80% confluent, they were collected using 11 mM EDTA (pH 7.6). BMDMs were resuspended in D10 for further experiments.

### AC generation

Thymocytes (Jurkats) were subjected to UV light for 20 min in a 10-cm dish in sterile PBS. Cells were collected, and media was changed to D10 media followed by a 2-h incubation at 37°C to allow for cells to become apoptotic. Cells were collected and centrifuged at 400 × *g* for 5 min. Cells were resuspended in 2 × 10^−6^ M PKH26 for 3 min. Staining was stopped by adding equivalent volumes of FBS.

### Mitochondrial bioenergetics

BMDMs collected from lipin-1^mEnzy^KO, lipin-1^m^KO, and littermate control mice were seeded at a density of 150,000 cells per well on XF24 cell culture microplates and allowed to incubate for 4 h. Experiments were conducted in XF assay medium containing 25 mM glucose, 2 mM l-glutamine, and 1 mM sodium pyruvate and analyzed using a Seahorse XFe 24 extracellular flux analyzer (Agilent Technologies). Where indicated, the following were injected: ATP-synthesis inhibitor oligomycin (Oligo; 1 μM), carbonyl cyanide 4-(trifluoromethoxy)phenylhydrazone (FCCP) (2 μM) to uncouple ATP synthesis, rotenone (100 nM) to block complex I, and antimycin A (1 μM) (Sigma-Aldrich) to block complex III. Oxygen consumption rate (OCR) was analyzed using Wave Desktop software (Agilent Technologies).

### Palmitate use

BMDMs collected from lipin-1^mEnzy^KO, lipin-1^m^KO, and littermate control mice were seeded at a density of 150,000 cells per well on XF24 cell culture microplates and allowed to incubate for 4 h. Macrophages were treated with 40 ng/ml IL-4 for 1.5 h. Media was then changed to nutrient-restricted media containing Agilent base media (100840-000) supplemented with 0.5 mM pyruvate (HyClone) and 0.5 mM GlutaMAX (35050-061; Thermo Fisher Scientific). Cells were then pretreated with 2 mM 2-deoxy-d-glucose (D6134; Sigma-Aldrich) for 15 min prior to the assay and maintained throughout. Directly before analysis, 100 μM palmitate was added, and OCR was assayed via the Seahorse extracellular flux analyzer (Agilent).

### MerTK receptor staining

Zymosan (0.1 mg) was injected i.p. into lipin-1^m^KO and littermate control mice and allowed to incubate for 6 d. Mice were then sacrificed, and the peritoneal lavage was collected in FACS wash buffer (1% BSA, 1 mM EDTA, and 0.1% sodium azide in PBS). A total of 500,000 isolated peritoneal cells were blocked with CD16/CD32 (1:200) (14-0161-86; eBioscience) for 20 min. Cells were incubated with PECy7-conjugated CD11b (1:4000) (25-0112-81, clone M1/70; eBioscience), AF700-conjugated anti-CD45.2 (1:2000) (109821, clone 104; BioLegend), FITC-conjugated anti-Ly6G (1:800) (551460, clone 1A8; BD Biosciences), PECy5-conjugated anti-F4/80 (1:400) (15-4801-80, clone BM8; Invitrogen), and PE-conjugated anti-MerTK Ab (1:500) (151506; BioLegend) for 30 min in the dark. Cells were spun at 400 × *g* for 5 min and resuspended in FACS fix (1% paraformaldehyde in FACS wash buffer). Appropriate F Minus One Controls were used to identify positive staining. Compensation controls (Comp Bead, 01-2222-42; Invitrogen) were used to exclude spectral overlap. Flow cytometry was performed using Quanteon flow cytometer (Acea Biosciences). Data analysis was performed using FCS express (Denovo Software) and NovoExpress (Acea Biosciences).

### Central carbon analysis

BMDMs from lipin-1^m^KO and littermate control mice were seeded at a density of 1 × 10^6^/ml into Falcon tubes (60818-500; VWR). Cells were either treated with or without IL-4 (40 ng/ml) for 4 h. Cells were then collected and spun at 400 × *g* for 5 min. Cell pellets were sent to Creative Proteomics (Shirley, NY) for central carbon analysis.

### Lipid uptake

Five hundred microliters of BMDMs from lipin-1^mEnzy^KO, lipin-1^m^KO, and littermate control mice were seeded at a density of 1 × 10^6^/ml in HBSS (SH30588.01; Thermo Fisher Scientific) containing 25 mM glucose into Falcon tubes (60818-500; VWR). BODIPY-labeled palmitate (D3821; Thermo Fisher Scientific) (100 μM) was added to macrophages and allowed to incubate for 1 h at either 37°C or 4°C. Cells were then washed and resuspended in FACS wash, and mean fluorescent intensity (MFI) was analyzed via flow cytometry.

### In vivo efferocytosis

Lipin-1^mEnzy^KO, lipin-1^m^KO, and littermate control mice were injected i.p. with 0.1 mg of zymosan. Six days after zymosan injection, 4 million PKH26-labeled AC were injected into the peritoneal cavity of the mice and allowed to incubate for 45 min. Mice were then sacrificed, peritoneal lavages were collected, and isolated cells were blocked and stained with PECy7-conjugated anti-CD11b (1:4000) (25-0112-81, clone M1/70; eBioscience), AF700-conjugated anti-CD45.2 (1:2000) (109821, clone 104; BioLegend), FITC-conjugated anti-Ly6G (1:800) (551460, clone 1A8; BD Biosciences), and PECy5-conjugated anti-F4/80 (1:400) (15-4801-80, clone BM8; Invitrogen). Lavages were then analyzed via flow cytometry.

### In vitro continuing efferocytosis

A total of 5 × 10^5^ BMDMs from lipin-1^m^KO and littermate control mice were seeded into Falcon tubes. PK26-labeled AC were added at a 4:1 (AC/macrophage) ratio and allowed to incubate for 2 h. Cells were washed to remove unbound AC, then incubated with PKH67-labeled AC (4:1) for 45 min. Cells were washed and stained with PECy7-conjugated anti-CD11b (1:4000) and PECy5-conjugated anti-F4/80 (1:400). Flow cytometry was performed to determine the ability of the macrophages to eat multiple AC.

### Statistical analysis

GraphPad Prism 5.0 (La Jolla, CA) was used for statistical analyses. A Student *t* test analysis was used for comparison between two data sets. Area under the curve analysis followed by a one-way ANOVA was performed on palmitate and continuing efferocytosis Seahorse data. All other statistical significance was determined using a one-way ANOVA with a Dunnett posttest. All in vivo and in vitro experiments were performed a minimum of three times. Figure legends provide specific details for each data set.

## RESULTS

### Lipin-1 is required for increased metabolic activity during lipid catabolic states

**FIGURE 1.**
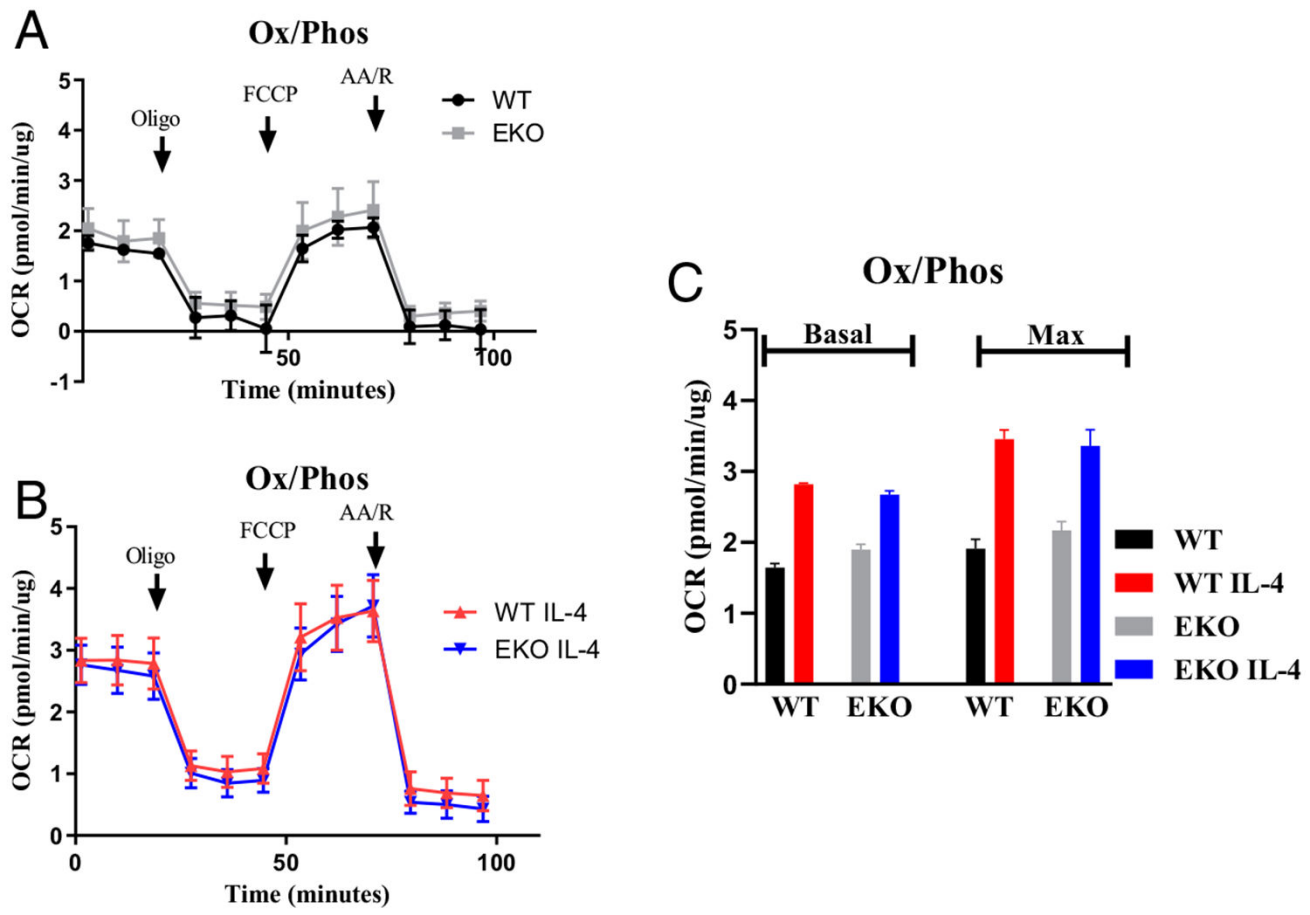
Lipin-1 enzymatic activity does not contribute to oxidative phosphorylation. BMDMs isolated from WT and EKO mice were treated with or without 40 ng/ml IL-4 for 4 h. Oxygen consumption was analyzed via Seahorse extracellular flux analyzer. (**A**) Untreated WT and EKO macrophages. (**B**) WT and EKO BMDMs treated with IL-4. (**C**) Basal and maximal (FCCP) OCR of WT and EKO BMDMs treated with and without IL-4. Graphed data represents mean OCR with SEM. *n* = 3.

**FIGURE 2.**
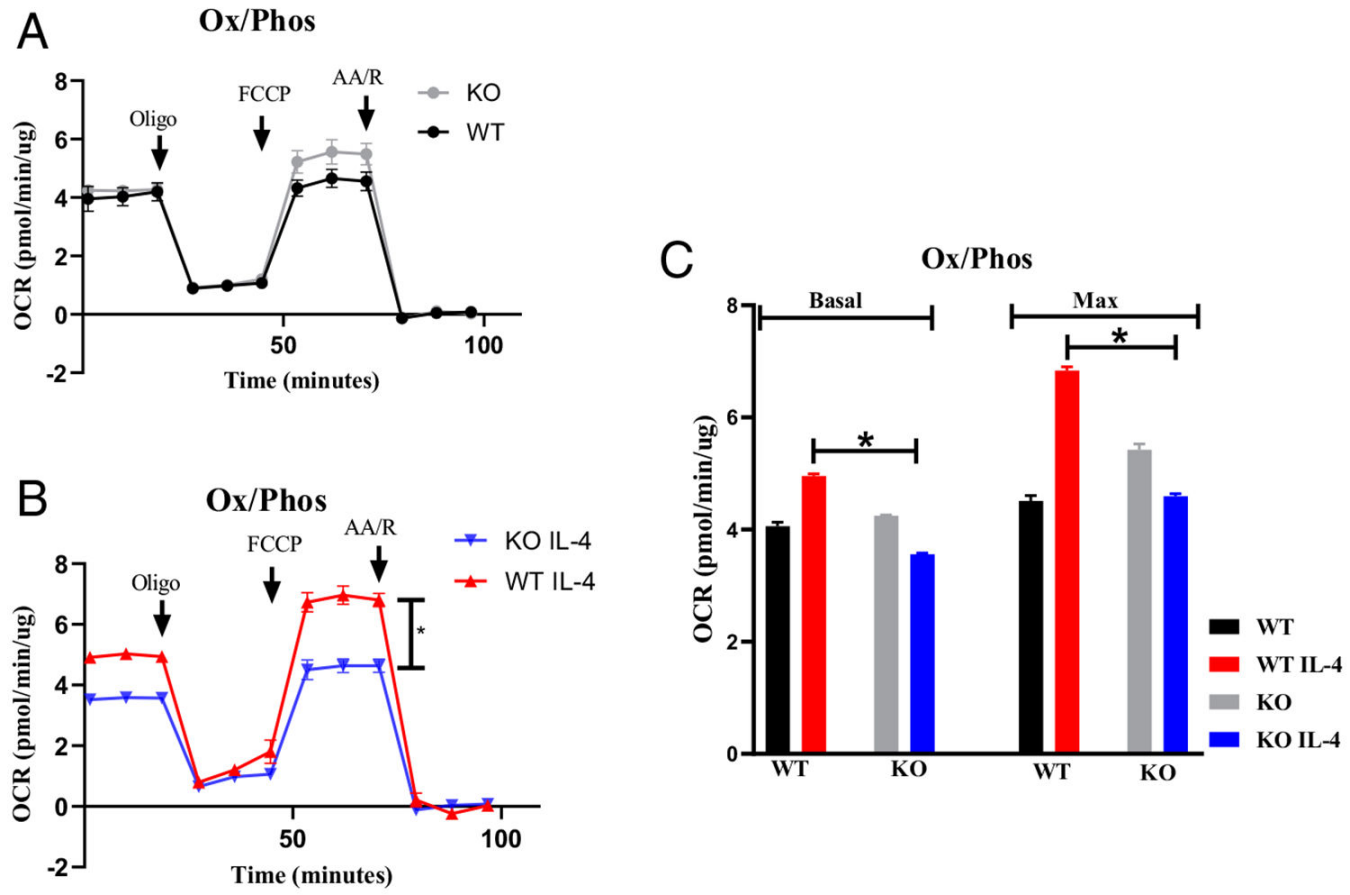
Lipin-1 transcriptional coregulator function contributes to oxidative phosphorylation. BMDMs isolated from WT and KO mice were treated with or without 40 ng/ml IL-4 for 4 h. Oxygen consumption was analyzed via Seahorse extracellular flux analyzer. (**A**) Untreated WT and KO macrophages. (**B**) WT and EKO BMDMs treated with IL-4. (**C**) Basal and maximal (FCCP) OCR of WT and EKO BMDMs treated with and without IL-4. *n* = 3. Graphed data represent mean OCR with SEM. *p ≤ 0.5.

Lipin-1 enzymatic activity promotes macrophage proinflammatory responses (18, 19), and we have recently demonstrated that lipin-1 transcriptional coregulatory activity contributes to IL-4 elicited gene expression in macrophages (22). IL-4 stimulation polarizes macrophages toward a proresolving macrophage and elicits increased β-oxidation and oxidative phosphorylation (7). In hepatocytes and skeletal muscle cells, lipin-1 promotes β-oxidation and oxidative phosphorylation (11, 23). We wanted to investigate if lipin-1 promoted oxidative phosphorylation in macrophages in response to IL-4. We used BMDMs from lipin-1^mEnzy^KO (EKO), lipin-1^m^KO (KO), and littermate controls (wild type [WT]) to determine the contribution of the enzymatic activity and infer the contribution of the transcriptional coregulatory activity toward induction of oxidative phosphorylation. BMDMs from EKO, KO, and WT mice were stimulated with 40 ng/ml IL-4 for 4 h, and cellular bioenergetics were analyzed by Seahorse extracellular flux analyzer (Agilent Technologies). There were no differences in respiration between unstimulated WT and EKO macrophages (Fig. 1A, 1C). As expected, IL-4 stimulation increased OCR in WT BMDMs, and an equivalent increase in OCR was observed in EKO BMDMs (Fig. 1B, 1C). These data suggest that the enzymatic activity of lipin-1 is dispensable for IL-4–elicited oxygen consumption. We next tested if lipin-1 transcriptional coregulatory activity contributes to oxidative metabolism in macrophages. WT and lipin-1 KO macrophages were stimulated with IL-4 for 4 h, and cellular bioenergetics were analyzed. There was no difference in respiration between unstimulated WT and lipin-1 KO BMDMs (Fig. 2A, 2C). Lipin-1 KO BMDMs failed to increase OCR in response to IL-4 stimulation, as seen in the WT BMDMs (Fig. 2B, 2C). The inability of lipin-1–deficient macrophages to increase OCR in response to IL-4, whereas macrophages lacking only lipin-1 enzymatic activity do respond to IL-4, suggests that lipin-1 transcriptional coregulator function is required for oxidative phosphorylation within IL-4–stimulated macrophages.

### Lipin-1 regulates lipid use within macrophages

**FIGURE 3.**
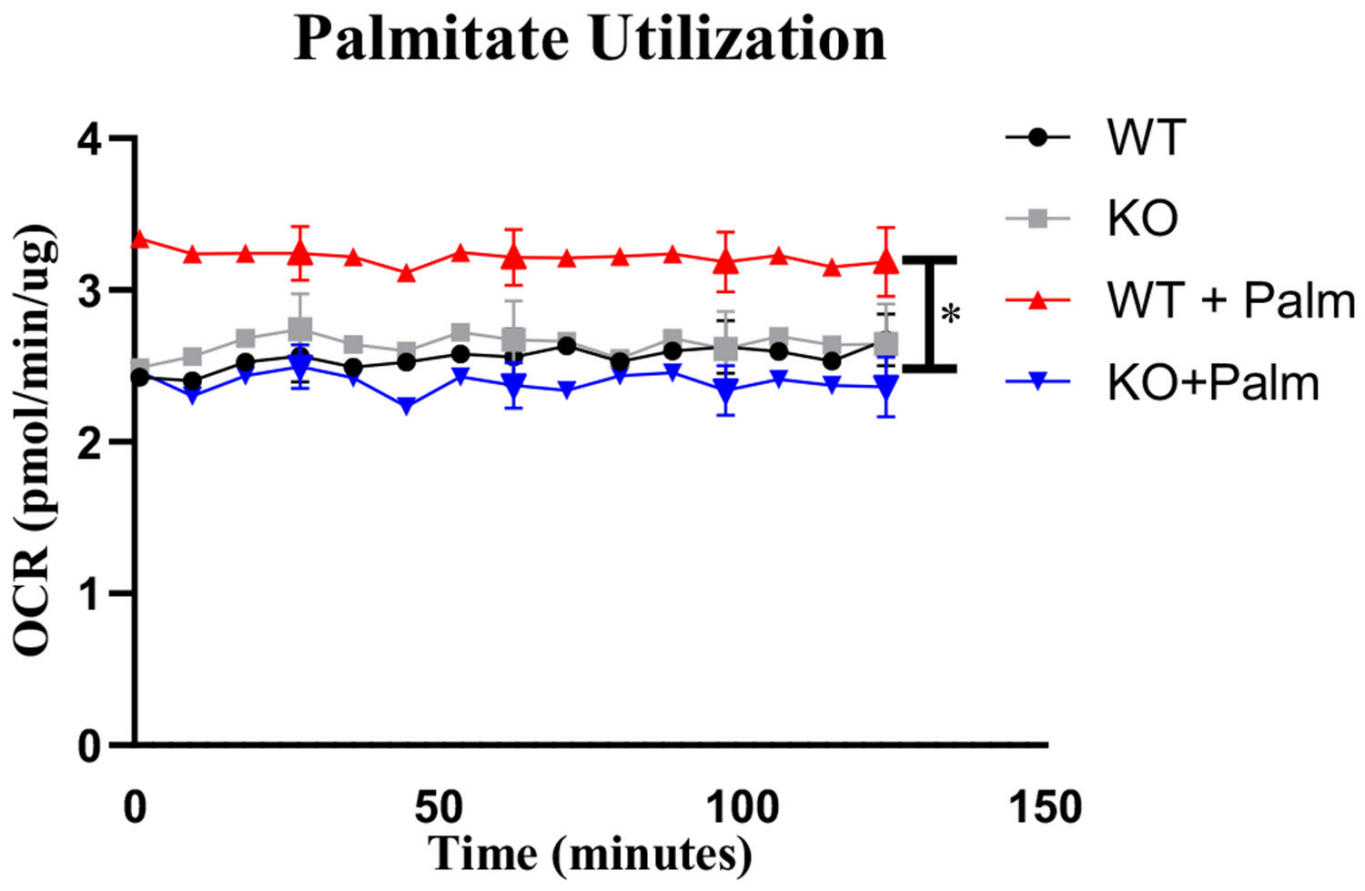
Lipin-1 is required for use of free fatty acids. BMDMs isolated from WT and KO mice were treated with 40 ng/ml for 1.5 h. Immediately before assay, 100 μM palmitate was added to requisite wells, and OCR was analyzed via Seahorse extracellular flux assay. Area under the curve was analyzed via one-way ANOVA. *n* = 3. Graphed data represent mean OCR with SEM. *p ≤ 0.5.

**FIGURE 4.**
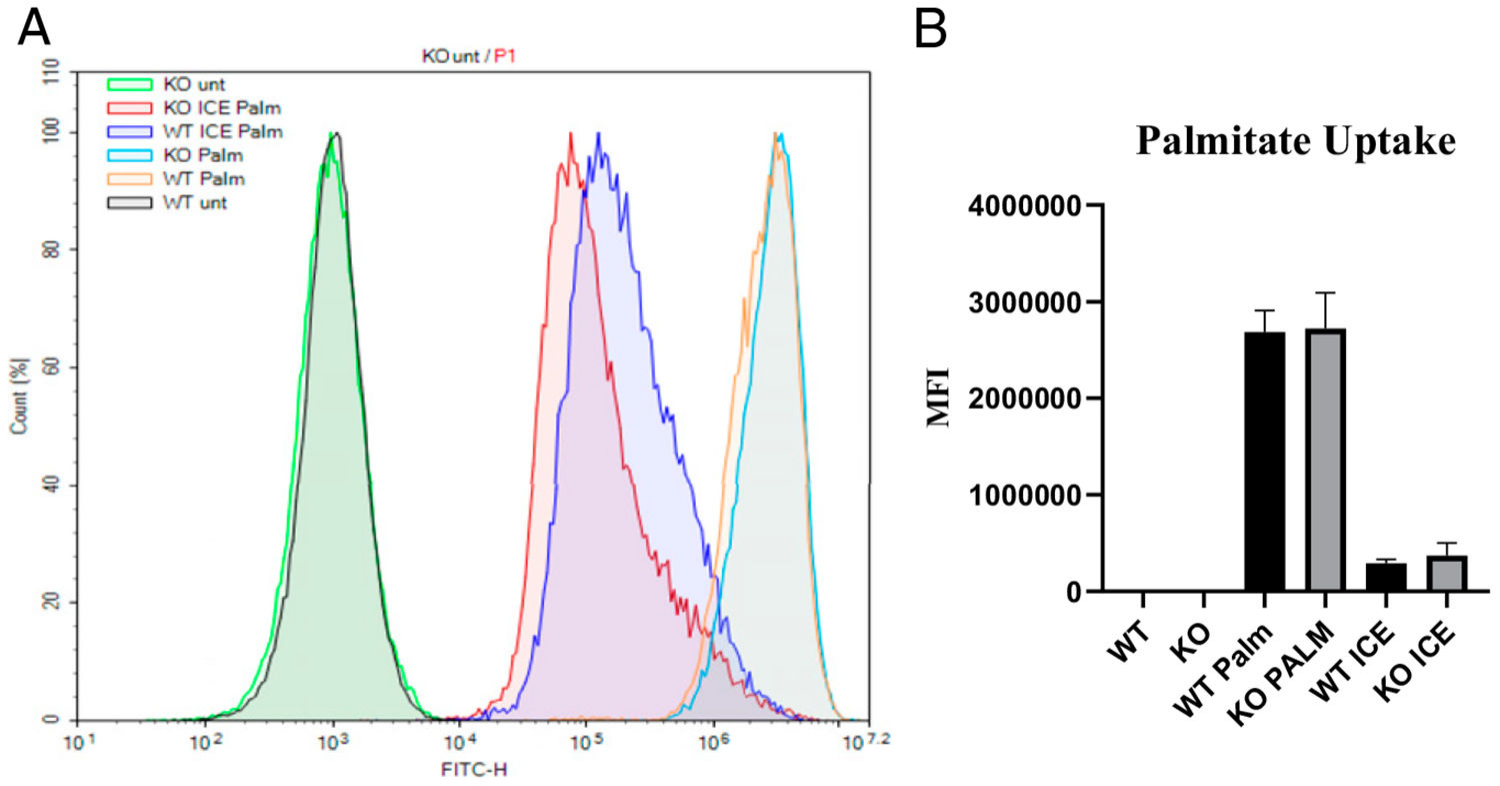
Lipin-1 does not regulate lipid uptake. BMDMs isolated from WT and KO mice were treated with 100 μM BODIPY palmitate for 1 h at either 37°C or at 4°C. BMDMs (CD11b^+^, F4/80^+^, FITC^+^, and Ly6G^−^) were analyzed via flow cytometry to determine MFI. (**A**) A representative histogram of WT and KO BMDMs treated with BODIPY palmitate. (**B**) Compiled MFI with SEM of WT and KO BMDMs treated with BODIPY palmitate. *n* = 3. Data were analyzed via a Student *t* test.

IL-4–stimulated macrophages shuttle fatty acids into the mitochondria to undergo β-oxidation and fuel oxidative phosphorylation (25). The loss of lipin-1 resulted in a failure of macrophages to increase oxygen consumption in response to IL-4, and we hypothesize this defect is due to an inability to break down lipids for energy, leading to reduced oxygen consumption. To determine if lipin-1–deficient macrophages can use lipids to promote oxygen consumption, we created an environment that is most favorable to using lipids for β-oxidation. We stimulated WT and lipin-1 KO BMDMs with IL-4 for 1.5 h to elicit β-oxidation in nutrient-(pyruvate and glutamine) restricted media. We inhibited glycolysis with the addition of 2-deoxy-d-glucose and added 100 μM palmitate to promote lipid-mediated oxygen consumption. OCR was measured via Seahorse XFe analyzer. We observed no difference in oxygen consumption between IL-4–treated WT and lipin-1 KO BMDMs not treated with palmitate (Fig. 3). The addition of palmitate to WT BMDMs significantly increased OCR, suggestive of β-oxidation and oxidative phosphorylation (Fig. 3). Treatment of lipin-1 KO BMDMs with palmitate failed to increase OCR as seen in WT BMDMs (Fig. 3). These data suggest that lipin-1 is critical for the breakdown and use of lipids in response to IL-4. A potential explanation for the inability of lipin-1 KO BMDMs to increase oxygen consumption when fed palmitate is a defect in uptake. To address iflipin-1 affects palmitic acid uptake, we treated WT and lipin-1 KO macrophages with IL-4 then fed them BODIPY-labeled palmitic acid and incubated for 1 h at either 4°C or 37°C to differentiate cellular binding from lipid uptake. Cells were analyzed via flow cytometry to determine MFI as a marker for palmitic acid uptake. There was no difference in MFI between WT and lipin-1 KO BMDMs (Fig. 4A, 4B). Taken together, these data suggest to us that the loss of lipin-1 from macrophages is not inhibiting lipid uptake but rather lipid breakdown by β-oxidation.

### Lipin-1 regulates cellular metabolism

**FIGURE 5.**
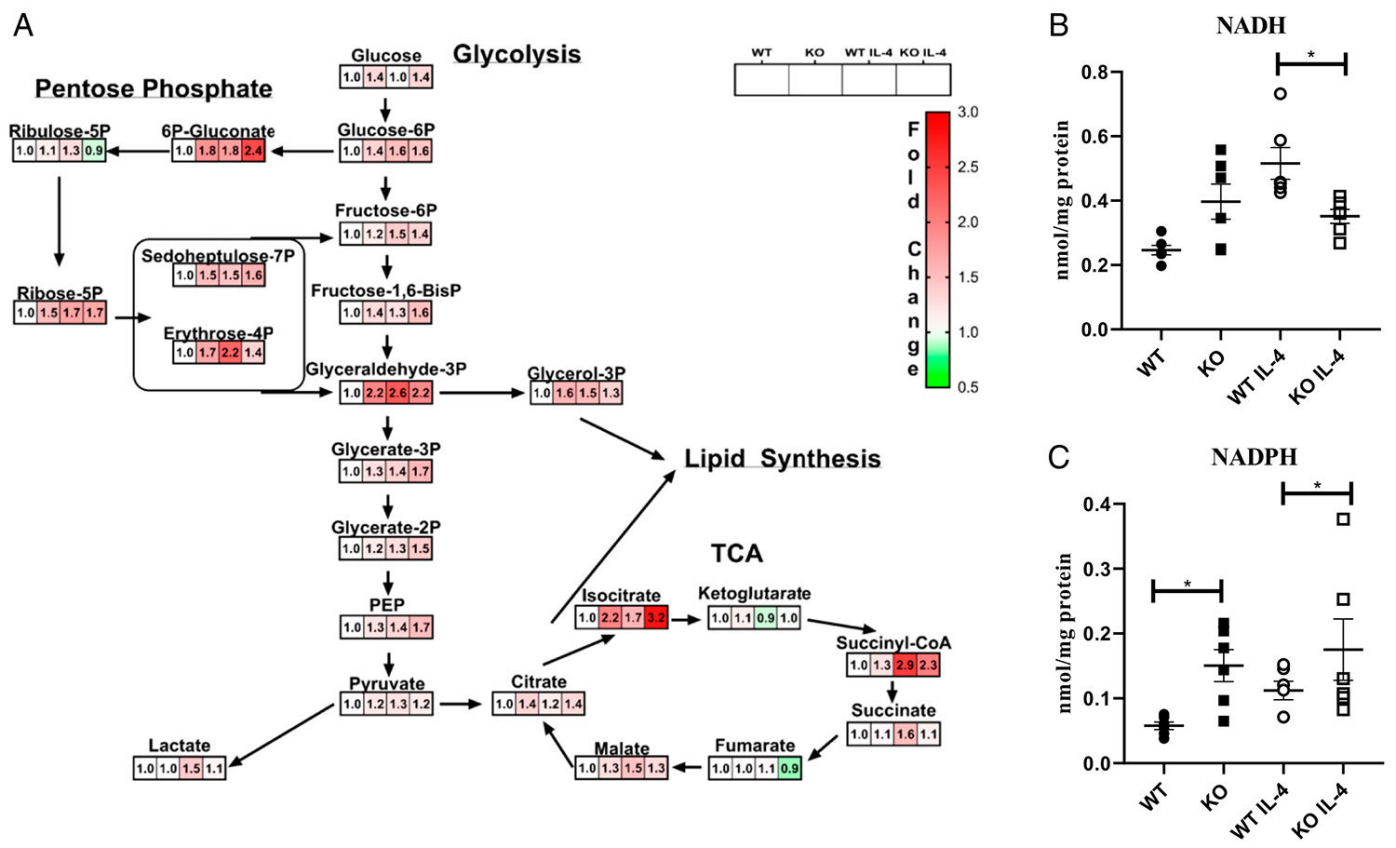
Lipin-1 regulates macrophage metabolism. Central carbon analysis of WT and KO BMDMs treated with and without 40 ng/ml IL-4 for 4 h (**A**). Values represent fold change compared with WT control. Quantitative analysis of NADH (**B**) and NADPH (**C**) levels in WT and KO BMDMs treated with and without 40 ng/ml IL-4 for 4 h. Graphed data represent mean metabolite concentration with SEM. Student *t* test was performed as statistical analysis. *n* = 6. *p ≤ 0.5.

Our data suggest that lipin-1 is required for the breakdown and use of lipid in response to IL-4 stimulation. Macrophages need to align individual metabolic pathways to effectively respond to stimuli. We wanted to understand the consequences of lipin-1 activity on immunometabolic responses in macrophages after stimulation with IL-4. WT and lipin-1 KO macrophages were stimulated with and without 40 ng/ml IL-4 for 4 h. Cell pellets were then sent to Creative Proteomics for central carbon analysis to determine the abundance of metabolites in major metabolic pathways, such as glycolysis, TCA cycle, and PPP. At baseline, lipin-1–deficient macrophages have an altered metabolism compared with WT macrophages. Lipin-1 KO BMDMs have multiple elevated glycolytic metabolites compared with WT BMDMs (Fig. 5A, Supplemental Fig. 1A). These data suggest that the loss of lipin-1 increases glycolysis. We also see an increase in metabolites within the PPP (Fig. 5A). Upregulation of the PPP can result in increased NADPH (Fig. 5A). There was also a significant increase in the TCA cycle metabolite isocitrate, an isomer of citrate, in lipin-1–deficient BMDMs compared with WT BMDMs (Fig. 5A, Supplemental Fig. 1B). This suggests that there is a break in the TCA cycle of lipin-1–deficient BMDMs, which leads to the accumulation of isocitrate in lipin-1–deficient BMDMs.

Stimulation of WT BMDMs with IL-4 resulted in a significant increase in many glycolytic and TCA cycle metabolites with no break in the TCA cycle that is consistent with a proresolving phenotype (Fig. 5A, Supplemental Fig. 1B). The alterations in metabolism in lipin-1–deficient BMDMs at baseline were exacerbated upon stimulation with IL-4. IL-4 stimulation of lipin-1 KO macrophages resulted in a significant increase in isocitrate, with a decrease in the TCA metabolite succinate (Fig. 5A, Supplemental Fig. 1B). These data suggest a break in the TCA cycle that is phenotypically similar to proinflammatory macrophages, which have decreased β-oxidation and oxidative metabolism (25). The TCA cycle generates electron carriers (NADH) that are used by the electron transport chain to generate ATP as part of oxidative phosphorylation. The loss of lipin-1 resulted in a significant decrease in NADH levels compared with WT macrophages (Fig. 5B), further supporting a break in the TCA cycle of lipin-1 KO macrophages. Lipin-1–deficient macrophages have an increase in the initial metabolites of the PPP, 6P-gluconate. The conversion of glucose-6P to 6P-gluconate generates NADPH, which can be used in lipid synthetic pathways (Fig. 5A). The increase in initial metabolites of the PPP is further supported by significantly elevated NADPH levels (Fig. 5C). These results suggest that lipin-1 regulates cellular metabolism in macrophages both basally and during IL-4 stimulation. Complete central carbon analysis can be found in Supplemental Table I.

### Lipin-1 promotes efferocytosis

**FIGURE 6.**
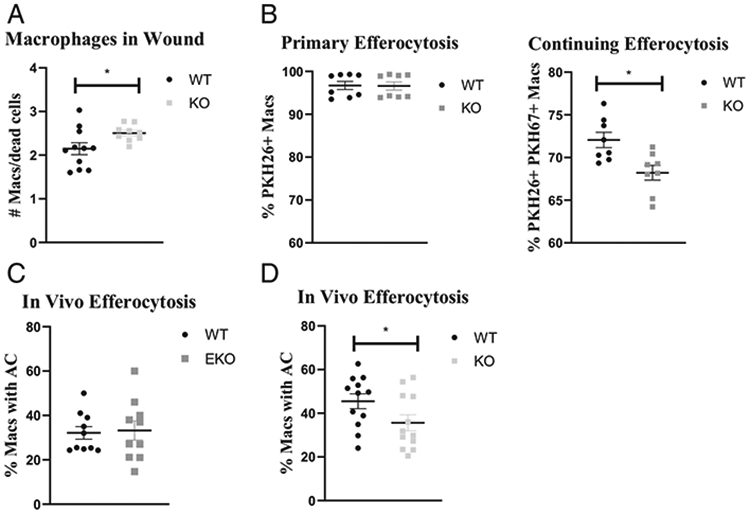
Lipin-1 contributes to efferocytosis. (**A**) WT and KO mice subjected to an excisional wound-healing model. Number of macrophages/dead cells was analyzed via flow cytometry. (**B**) BMDMs from WT and KO mice were subjected to a dual-label in vitro model of continuing efferocytosis, and percentage of macrophages (CD11b^+^ and F4/80^+^) that took up an initial event (primary) and multiple apoptotic bodies (continuing) were analyzed via flow cytometry. WT, EKO (**C**), and KO (**D**) mice were subjected to a zymosan model of peritonitis followed by peritoneal injection of PHK26-labeled AC. Percentage of macrophages (CD11b^+^, F4/80^+^, PKH26^+^, and Ly6G^−^) with labeled AC were analyzed via flow cytometry. Graphed data represent mean uptake with SEM. Student *t* test was used to analyze data. *n* = 8–12. *p ≤ 0.5.

Macrophages mediate tissue homeostasis by the clearance of dead and dying cells by a process termed efferocytosis. Engagement of AC elicits proresolving responses from macrophages (8). Recent studies have demonstrated that β-oxidation of AC-derived lipids is necessary for IL-10 production (proresolving cytokine) and subsequent wound healing in a mouse model of myocardial infarction (8). Defects in efferocytosis lead to unresolved inflammation that perpetuates disease pathogenesis, as seen in atherosclerosis, obesity, diabetes, and autoimmunity (26-28). We have demonstrated that mice lacking myeloid-associated lipin-1 have increased necrotic cores within atherosclerotic plaques (C.M.R.B., doi: 10.1101/2020.06.02.130096) and have delayed wound closure (22). Inhibition of efferocytosis can contribute to increased necrotic core formation during atherosclerosis and delayed wound closure (29, 30). We re-examined data collected from Chandran et al. (22) of the number of macrophages to the number of dead cells within wounds of lipin-1^m^KO mice and littermate controls. Although there was no difference in the total number of dead cells in the wound between lipin-1^m^KO mice and littermate controls, we observed a greater ratio of macrophages/dead cells within wounds of lipin-1^m^KO mice as compared with littermate controls (Fig. 6A). When cell death is extensive, as seen in atherosclerotic plaques or within wounds, successive uptake of ACs by proresolving macrophages termed “continuing efferocytosis” is necessary. This may suggest that macrophages from lipin-1^m^KO mice may have a defect in either efferocytosis or continuing efferocytosis.

We decided to determine if lipin-1 is required for efferocytosis/continuing efferocytosis. To examine both primary efferocytosis (engulfment of a single AC) and continuing efferocytosis (engulfment of a second cell), we performed an in vitro continuing efferocytosis experiment. We selected incubation times based on previous work investigating continuing efferocytosis (31). Briefly, PKH26-labeled AC were added to WT and KO BMDMs at a 4:1 ratio and allowed to incubate for 2 h. Two hours allows the macrophages to engulf and begin to degrade AC-derived macromolecules from the primary efferocytic event (32). Cells were washed, and PKH67-labeled AC were added to the BMDMs at a 4:1 ratio and allowed to incubate for 45 min, which is sufficient time for engulfment of a second AC (continuing efferocytosis). Cells were then stained, and the percentage of PKH26^+^ PKH67^+^ macrophages was quantified via flow cytometry. Although there was no difference in the ability to take in one AC (Fig. 6B), there was a significant reduction in the uptake of multiple AC (continuing efferocytosis) in lipin-1–deficient macrophages compared with WT controls (Fig. 6B). Although the difference in continuing efferocytosis between WT and KO BMDMs was modest, typically these modest differences are more exaggerated in vivo. This has been reported in other studies investigating efferocytosis (31). To examine efferocytosis in vivo, we subjected lipin-1^mEnzy^KO, lipin-1^m^KO, and littermate control mice to a zymosan model of inflammation. Briefly, mice were injected i.p. with 0.1 mg of zymosan. The inflammatory response was allowed to continue for 6 d to establish a proresolving milieu (33). Labeled AC were injected into the peritoneal cavity for 45 min. Peritoneal lavages were collected, and percentages of macrophages with labeled AC were quantified via flow cytometry. Lipin-1^m^KO mice had a significant reduction in the percentage of macrophages with AC compared with both WT and lipin-1^mEnzy^KO (Fig. 6C, 6D). Macrophages in the peritoneal cavity likely encounter AC prior to the addition of labeled AC, suggesting to us a defect in secondary efferocytosis as observed in vitro. Lipin-1 transcriptional coregulatory function regulates PPARs, which have been shown to increase efferocytic receptors such as MerTK (11,34). A reduction in efferocytosis receptors in lipin-1 KO macrophages could explain the defect in efferocytosis. To determine if the loss of lipin-1 results in an alteration in cell surface expression of MerTK, we stained peritoneal lavages for macrophages (CD11b and F4/80) and analyzed the presence of MerTK via flow cytometry. There was no significant difference in cell surface expression of MerTK in WT and lipin-1 KO macrophages (Supplemental Fig. 2), suggesting the defect in efferocytosis is not due to the lack of MerTK. These data demonstrate that lipin-1 enzymatic activity is not involved in efferocytosis and suggest the transcriptional coregulatory activity promotes continuing efferocytosis in macrophages.

**FIGURE 7.**
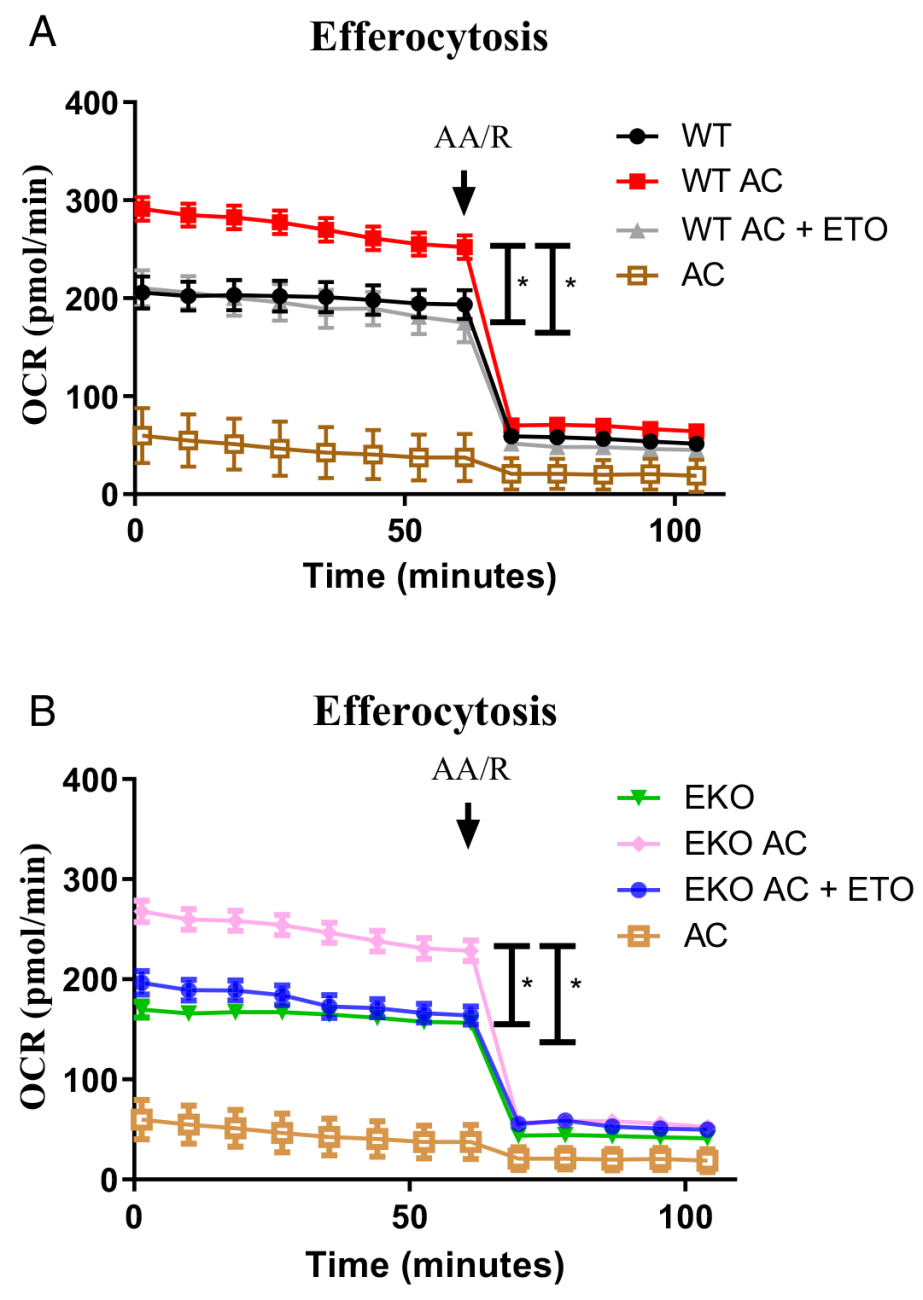
Lipin-1 enzymatic activity does not contribute to AC-derived lipid use. OCR of WT (**A**) and EKO (**B**) BMDMs pretreated with and without 40 μM etomoxir for 15 min followed by the addition of AC at a 4:1 ratio. Graphed data represent mean OCR with SEM. *n* = 3. *p ≤ 0.5.

**FIGURE 8.**
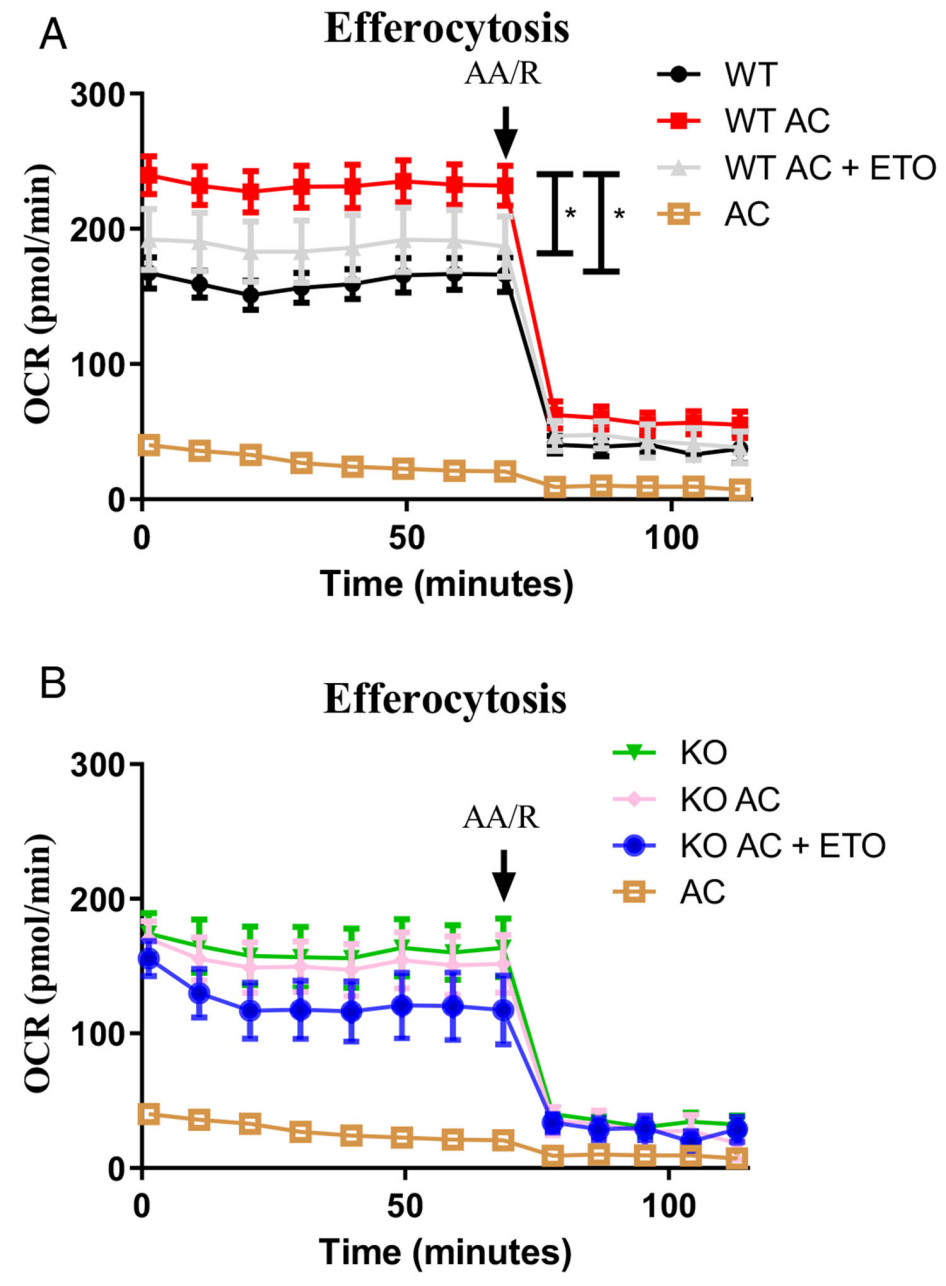
Lipin-1 transcriptional coregulator function is required for degradation of AC-derived lipids. OCR of WT (**A**) and KO (**B**) BMDMs pretreated with and without 40 μM etomoxir for 15 min followed by the addition of AC at a 4:1 ratio. Graphed data represent mean OCR with SEM. *n* = 3. *p ≤ 0.5.

We have shown that lipin-1 is required for increased OCR in response to free fatty acids, but we wanted to know if lipin-1 regulates β-oxidation in response to AC during efferocytosis. BMDMs from WT, lipin-1^mEnzy^KO, and lipin-1^m^KO mice were either pretreated with or without etomoxir to prevent fatty acid transport into the mitochondria followed by the addition of AC to the macrophages at an AC/BMDM ratio of 4:1. AC were incubated with BMDMs for 30 min prior to the start of Seahorse analysis. This is sufficient time for differences in OCR due to β-oxidation of AC-derived lipids to be observed. After 1 h of OCR readings, antimycin A and rotenone were added to the cells to inhibit electron import into the electron transport chain. This ensures that the observed oxygen consumption is specific to the mitochondria. OCR was then analyzed via Seahorse XFe analyzer (Agilent Technologies). WT and lipin-1 EKO macrophages significantly increased OCR in response to AC, with a significant reduction with pretreatment of etomoxir (Fig. 7A, 7B). This suggests that the increase in oxidative metabolism is due to the catabolism of AC-derived lipids in both WT and EKO macrophages. However, lipin-1 KO macrophages failed to respond to AC compared with WT macrophages, with no significant difference in OCR with pretreatment of etomoxir (Fig. 8A, 8B). Etomoxir treatment of unstimulated (no AC) WT, lipin-1 EKO, and lipin-1 KO macrophages allows us to determine basal lipid use. There was a significant reduction in OCR in WT, lipin-1 EKO, and lipin-1 KO macrophages with treatment with etomoxir alone (Supplemental Fig. 3A, 3B). These data suggest that there is no defect in basal β-oxidation but an inability to undergo β-oxidation upon lipid loading in our lipin-1 KO macrophages. Together, these data imply that the transcriptional coregulatory function of lipin-1 is required for β-oxidation of AC-derived lipids during efferocytosis, whereas the enzymatic activity of lipin-1 is not required.

## DISCUSSION

Proresolving macrophages undergo drastic metabolic changes to effectively respond to stimuli. These proresolving macrophages significantly increase β-oxidation and oxidative phosphorylation, and perturbations in these pathways are deleterious to macrophage function (7). We have previously demonstrated that lipin-1 contributes to atherosclerotic progression and macrophage polarization toward a proresolving phenotype in response to IL-4 (19, 22). Using our lipin-1^mEnzy^KO, lipin-1^m^KO, and littermate control mice, we provide evidence that lipin-1 transcriptional coregulator function is critical for induction of β-oxidation in response to proresolving stimuli. We also provide evidence that the loss of lipin-1 transcriptional coregulatory activity also impacts efferocytic function of macrophages, which may contribute to impairment in disease resolution. These results are some of the first (to our knowledge) to implicate lipin-1 activity in regulating continuing efferocytosis.

IL-4 stimulation, as well as efferocytosis, leads to activation of transcription factors such as STAT6 and PPARs to drive expression of genes involved in fatty acid transport and β-oxidation (34, 35). Lipin-1 binds to and augments PPAR activity in a variety of tissues (11, 12). The removal of exons 3 and 4 of lipin-1 in our lipin-1^mEnzy^KO mice results in truncated lipin-1 that lacks enzymatic activity but retains the ability of lipin-1 to bind to transcription factors such as PPARα and PPARγ (19, 23, 36, 37). Lipin-1^mEnzy^KO macrophages had equivalent levels of OCR in response to both IL-4 stimulation and AC. Removal of exon 7 from lipin-1 in our lipin-1^m^KO mice causes a missense protein, leading to loss of lipin-1 and both activities (23). BMDMs from lipin-1^m^KO mice showed a significant reduction in OCR in response to IL-4 and ACs. Loss of lipin-1 did not result in reduction of either free lipid or AC, suggesting that the defect in β-oxidation is not due to the inability of the macrophages to take in the lipid or perform primary efferocytosis. Further studies will investigate trafficking and use of the lipid within the macrophage after either free fatty acid feeding or AC feeding.

Macrophage metabolism is directly connected to their polarization state and role during the inflammatory response (7). Central carbon analysis of WT and lipin-1 KO macrophages untreated and treated with IL-4 revealed an overall increase in glycolysis in lipin-1–deficient macrophages. Interestingly, the loss of lipin-1 led to a significant decrease in lactate, with no alteration in pyruvate. Lactate can be shuttled into the mitochondria to be converted to pyruvate to fuel the TCA cycle (38). Untreated and IL-4–treated lipin-1 KO macrophages have an accumulation of isocitrate, an isomer of citrate. Downstream TCA cycle metabolites after isocitrate were reduced in lipin-1 KO macrophages compared with WT macrophages. This is further supported by a decrease in NADH levels as well as a significant decrease in OCR in lipin-1–deficient macrophages. Isocitrate is an isomer of citrate, which can be exported out of the mitochondria to be used in lipid synthetic pathways, similar to proinflammatory macrophages (39). Lipin-1–deficient macrophages have an increase in the initial metabolites of the PPP, resulting in increased NADPH levels. NADPH is used by the enzymes of lipid synthetic pathways as well as for redox potential within the cell (40). Glycerol-3-phosphate is used as a backbone for glycerol lipid synthesis and is elevated in the lipin-1 KO macrophages (Supplemental Table I). Altogether, the loss of lipin-1 results in a metabolic profile that is supportive of lipid synthesis, rather than lipid catabolism. Interestingly, previous studies have shown that de novo lipid synthesis, such as ceramides, is detrimental to efferocytosis, supporting data shown in our current study (41). The inability to undergo β-oxidation of exogenous lipid, together with the metabolic profile of lipin-1 KO macrophages, may suggest that the incoming free fatty acids are being used for de novo lipid synthesis.

Efferocytosis is a complex process that leads to the accumulation of macromolecules within the macrophage that need to be processed and cleared. AC-derived arginine is metabolized, leading to RAC1 activation and further actin polymerization to allow for uptake of multiple AC (31). Lipid is a major component of AC. Mitochondria are critical for the process of efferocytosis (8, 42). Mitochondria must undergo fission to mobilize around the engulfed AC to allow for continuing efferocytosis (42). This is presumably to accept incoming fatty acids for β-oxidation. β-Oxidation of AC-derived lipids is required for anti-inflammatory cytokine production and wound healing in a mouse model of myocardial infarction (8). These studies eloquently show that β-oxidation contributes to functional outcomes of efferocytosis during disease resolution. We demonstrated that loss of lipin-1 did not impact primary efferocytosis but rather secondary or continuing efferocytosis. Furthermore, we demonstrate a lack of increased β-oxidation in response to AC stimulation. We speculate that during primary efferocytosis, β-oxidation is not required for engulfment of AC. Our work with zymosan particles supports this idea as baseline engulfment of these particles is not impacted by loss of lipin-1 in macrophages, but there is a reduction in zymosan particle uptake during IL-4–mediated enhancement (22). We believe that following engulfment of the first AC, there is an abundance of lipid that must be processed and degraded via β-oxidation that is mediated by lipin-1 before the macrophage is able to take in another AC (continuing efferocytosis). Currently the mechanism connecting β-oxidation is unknown, but there are several potential hypotheses. There may be a limit on the amount of engulfed lipid that macrophage can handle, which must be cleared prior to accepting a second AC. We feel this is unlikely based on the amount of lipid macrophage foam cells engulf. There is the potential that β-oxidation supplies energy more efficiently than other mechanisms, allowing for continuing efferocytosis. There maybe downstream metabolites generated from the degradation of AC-derived lipid that mediate signaling cascades to promote continuing efferocytosis, similar to Arginine-mediated enhancement of continuing efferocytosis (31).

Our data suggest that not only is the transcriptional coregulatory activity of lipin-1 required for increased oxidative phosphorylation and lipid catabolism in macrophages in response to proresolving stimuli, but it is required for functional outcomes of that stimulation as well. We show that lipin-1 is required for efficient continuing efferocytosis. Mice lacking myeloid-associated lipin-1 have reduced AC uptake in a zymosan model of efferocytosis. Furthermore, our previous data demonstrate that the loss of lipin-1 results in a reduction in proresolving/woundhealing polarization in response to IL-4 and defects in wound healing in vivo (22). We would propose that lipin-1 transcriptional coregulatory activity is critical to macrophage proresolving polarization. Further studies will investigate how lipin-1 transcriptional coregulatory activity primes β-oxidation in macrophages, if β-oxidation is required for macrophages to perform continuing efferocytosis, and how β-oxidation allows for continuing efferocytosis.

## Supplementary Material

Supplemental Figure

Supplemental Table

## Abbreviations used in this article:

AC: apoptotic cell
BMDM: bone marrow–derived macrophage
EKO: lipin-1^mEnzy^KO
FCCP: carbonyl cyanide 4-(trifluoromethoxy) phenylhydrazone
KO: lipin-1^m^KO
MFI: mean fluorescent intensity
OCR: oxygen consumption rate
PPP: pentose phosphate pathway
WT: wild type
