# Supplementary material for: Macrophage-Associated Lipin-1 Promotes β-Oxidation in Response to Proresolving Stimuli
Source: Immunohorizons. Author manuscript; Available in PMC 2020 Dec 16. (PMC7739271; doi:10.4049/immunohorizons.2000047)
Supplement: Supplemental Table [file NIHMS1651551-supplement-Supplemental_Table.pdf]

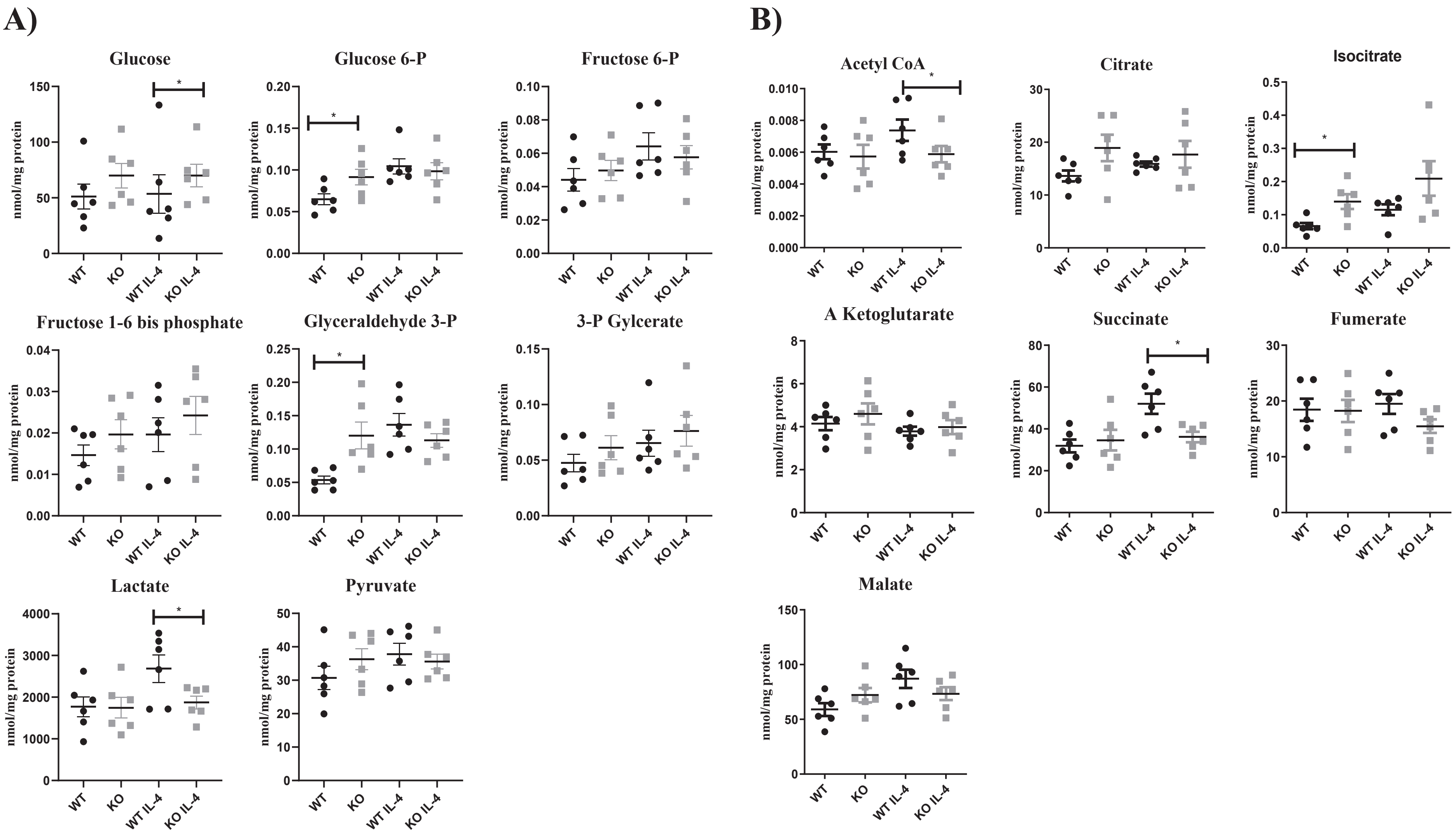

Supplemental figure 1: Lipin-1 coordinates metabolism within macrophages.

Central carbon analysis of glycolytic intermediates (A) and TCA cycle intermediates (B) from WT and KO BMDMs treated with and without 40 ng/mL IL-4 for 4 hours. Graphed data represents mean metabolite concentration with standard error of the mean. N=3. * indicate $p \leq 0.05$

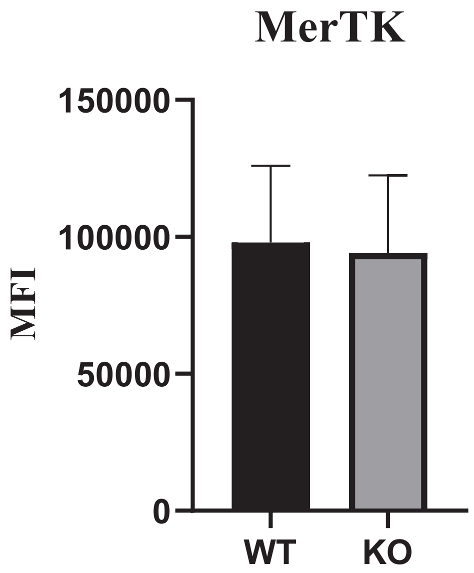

Supplemental figure 2: Lipin-1 does not regulate MerTK cell surface expression.

WT and KO mice were subjected to a zymosan model of peritonitis. Flow cytometry analysis of macrophage (CD11b+, F4/80+, MerTK+, and Ly6G-) MerTK was performed on peritoneal lavages. Graphed data represents mean MFI with standard error of the mean. N=3.

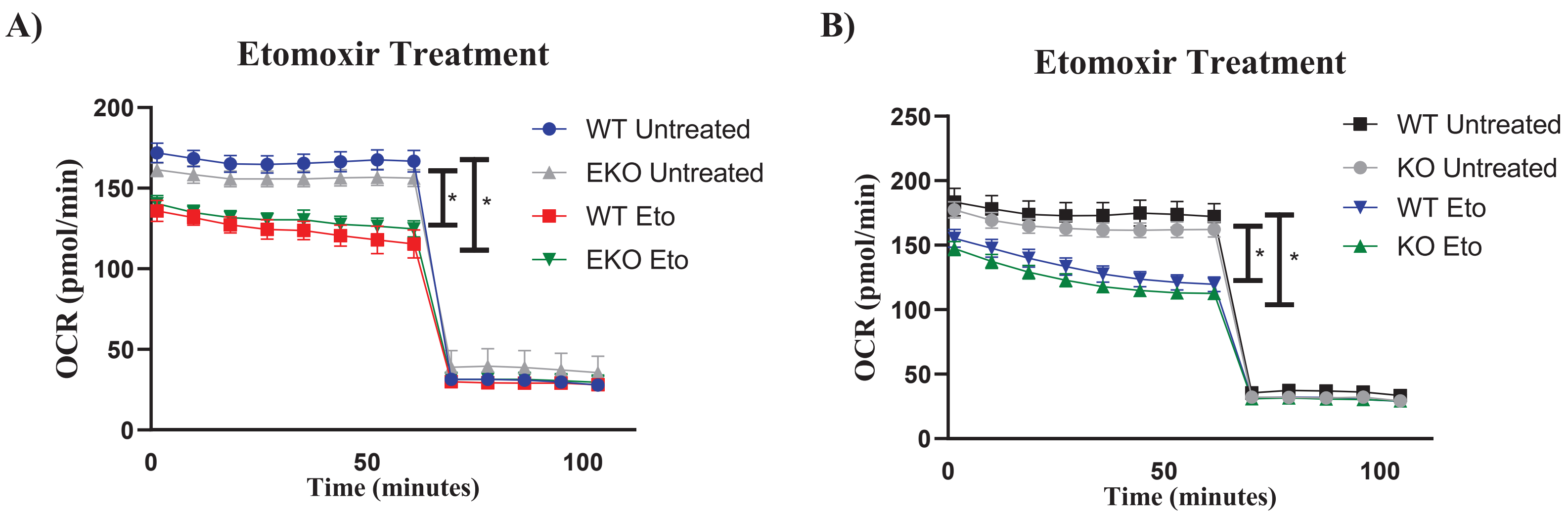

Supplemental figure 3: Equivalent levels of basal lipid utilization.

BMDMs from WT, EKO (A), and KO (B) mice were treated with and without etomoxir for 20 minutes. Seahorse analysis was performed to determine oxygen consumption rate (OCR). Graphed data represents mean OCR with standard error of the mean. N=3. * indicate $p \leq 0.05$
